# Mesoporous Carbon and Ceria Nanoparticles Composite Modified Electrode for the Simultaneous Determination of Hydroquinone and Catechol

**DOI:** 10.3390/nano9010054

**Published:** 2019-01-03

**Authors:** Dong Liu, Fan Li, Dezhong Yu, Junxia Yu, Yigang Ding

**Affiliations:** 1 School of Chemistry and Environmental Engineering, Wuhan Institute of Technology, Wuhan 430205, China; liudong1980@aliyun.com (D.L.); Lifan19951002@163.com (F.L.); yudezhongwh@163.com (D.Y.); yujunxia_1979@163.com (J.Y.); 2 Key Lab for Green Chemical Process of Ministry of Education, Wuhan Institute of Technology, Wuhan 430205, China; 3 State Key Laboratory of Oil and Gas Reservoir Geology and Exploitation, Southwest Petroleum University, Chengdu 610500, China

**Keywords:** mesoporous carbon and ceria nanoparticles composite, modified electrode, simultaneous determination, hydroquinone, catechol

## Abstract

In this work, a novel material that was based on mesoporous carbon and ceria nanoparticles composite (MC–CeNPs) was synthesized, and a modified electrode was fabricated. When compared with a bare glass electrode, the modified electrode exhibited enhanced electrocatalytic activity towards the simultaneous determination of hydroquinone (HQ) and catechol (CC), which is attributed to the large specific area and fast electron transfer ability of MC–CeNPs. Additionally, it exhibited linear response ranges in the concentrations of 0.5–500 µM and 0.4–320 µM for HQ and CC, with detection limits (S/N = 3) of 0.24 µM and 0.13 µM, respectively. This method also displayed good stability and reproducibility. Furthermore, the modified electrode was applied to the simultaneous determination of HQ and CC in tap and lake water samples, and it exhibited satisfactory recovery levels of 98.5–103.2% and 98–103.4% for HQ and CC, respectively. All of these results indicate that a MC–CeNPs modified electrode could be a candidate for the determination of HQ and CC.

## 1. Introduction

Hydroquinone (HQ) and catechol (CC) are two isomers of dihydroxybenzene and they are easily introduced into the environment during their extensive use as important raw materials and intermediates [1]. In addition, the United States Environmental Protection Agency (EPA) and the European Union (EU) have revealed that HQ and CC are important environmental pollutants in ecological systems, owing to the fact that they are extremely toxic to human health and ecology, even in very low concentration [2]. HQ and CC are similar in structure and properties and they often coexist in the environment, which makes their detection more difficult [3]. Accordingly, it is necessary to establish an efficient method for the simultaneous determination of these two isomers. Many methods have been developed for their determination, such as fluorescence [4], gas chromatography [5], high–performance liquid chromatography [6], chemiluminescence [7], and electrochemical methods [8]. In the last few years, electrochemical methods have attracted wide attention for their fast response, high sensitivity, low–cost, selectivity, and facile to use [9,10,11]. However, numerous challenges exist in the use of electrochemical methods for the simultaneous determination of HQ and CC. The two isomers have overlapped redox potentials on a conventional electrode [12], and another significant obstacle is that the relationship between the electrochemical signal and concentration might be nonlinear. Many excellent nanomaterials, such as Au@Pd/graphene [13], AuNPs–mesoporous silica [14], MWCNT/TiO_2_ [15], and carbon nano–fragments [16], have been used to modify electrodes to overcome these shortages, and the resulting electrodes have exhibited good detection limits. However, these materials have disadvantages in their complicated synthesis process and high cost. Thus, it is necessary to develop a novel material to detect HQ and CC.

Nanostructured ceria (CeO_2_) is often used as a catalytic medium due to its oxygen storage capacity via the Ce^4+^/Ce^3+^ redox reaction. It is also considered to be an ideal candidate for electrochemical sensors, due to its abundant active sites, oxygen vacancies, biocompatibility, and excellent catalytic performance [17]. However, ceria nanoparticles (CeNPs) tend to form aggregates that cause lower catalytic activity and worse stability [18]. In addition, poor conductivity hinders their application. Therefore, appropriate supporting substrates to anchor CeNPs are expected to enhance the dispersion and conductivity, and improve the catalytic activity.

Currently, some significant breakthroughs have been made concerning nanostructured carbon composite material in electrochemical sensors [19], catalysts [20,21,22,23], and supercapacitors [24]. Additionally, many ceria–carbon composites have been reported, such as CeO_2_/graphene [25], CeO_2_/g–C_3_N_4_ [26], and MWCNTs/CeO_2_ [27]. Mesoporous carbon (MC) is a kind of nanostructured porous carbon materials; it can be an excellent carrier for sensors because of its good conductivity, high porosity, high surface area, high corrosion resistance, and easy handling. When compared with other carbon materials, such as graphene [28] and carbon nanotubes [29], MC could have better electrocatalytic ability and electrochemical properties because of its unique pore structure, which is conducive to mass transfer [30,31]. However, few studies have investigated electrochemical sensors using ceria and MC. Thus, it is necessary to synthesis the composite material and investigate its electrochemical performance. 

**Scheme 1:**
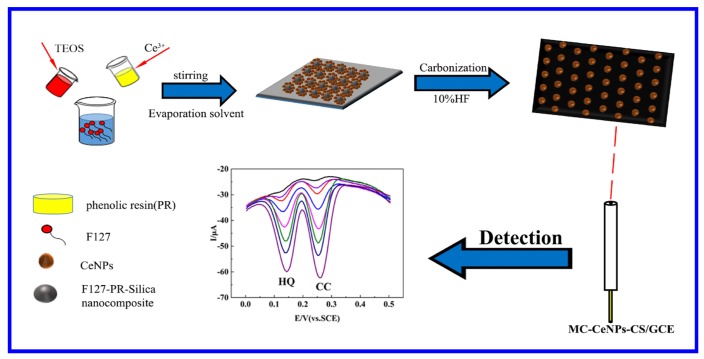
Fabrication of the mesoporous carbon and ceria nanoparticles composite (MC–CeNPs) modified electrode and the simultaneous detection of hydroquinone (HQ) and catechol (CC).

In this work, MC–CeNPs were synthesized via the evaporation induced self-assembly (EISA) approach and characterized by scanning electron microscopy, transmission electron microscope, X-ray diffraction, Raman spectra, nitrogen adsorption–desorption, and X-ray photoelectron spectroscopy. The obtained composite was dispersed in chitosan (CS) solution and dropped on a glassy carbon electrode (GCE) to prepare the sensitive sensor, which exhibited good potential for separation and differentiation for the simultaneous detection of HQ and CC. Scheme 1 exhibits the overall preparation of MC–CeNPs modified electrode. To our best knowledge, this is the first time that HQ and CC have been detected using mesoporous carbon and ceria nanocomposites. The sensing platform exhibited wide linear response ranges, low detection limits, and suitability for practical applications with real water samples due to the large specific area, excellent electrocatalytic performance, and fast electron transfer ability of the nanostructured composite. Furthermore, the selectivity and storage stability of the modified electrode were studied and found to have satisfactory results. 

## 2. Results and Discussion

### 2.1. Characterization of MC–CeNPs 

**Figure 1:**
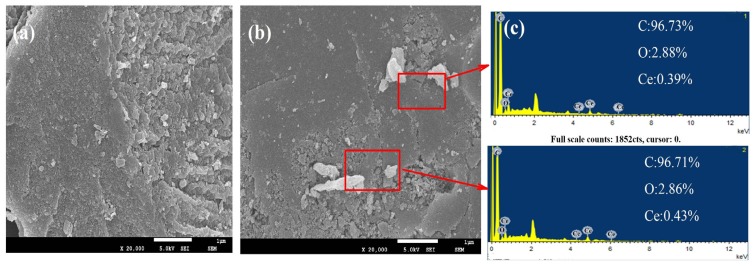
Scanning electron microscope (SEM) images of mesoporous carbon (MC) (**a**) and MC–CeNPs (**b**); energy dispersive X-ray detector (EDX) image of MC–CeNPs (**c**).

The surface morphology of the composite and its individual component was characterized using FESEM and it is shown in Figure 1 (each scale bar is 1 μM). As illustrated in Figure 1a, MC exhibited a layered structures with wrinkles. Figure 1b depicts that MC–CeNPs tend to form a more whole structure when compared with the MC, and few layers could be observed. Furthermore, the composition of elements was tested by EDX (Figure 1c). Only Ce, C, and O could be observed, suggesting that the MC–CeNPs were of high purity.

**Figure 2:**
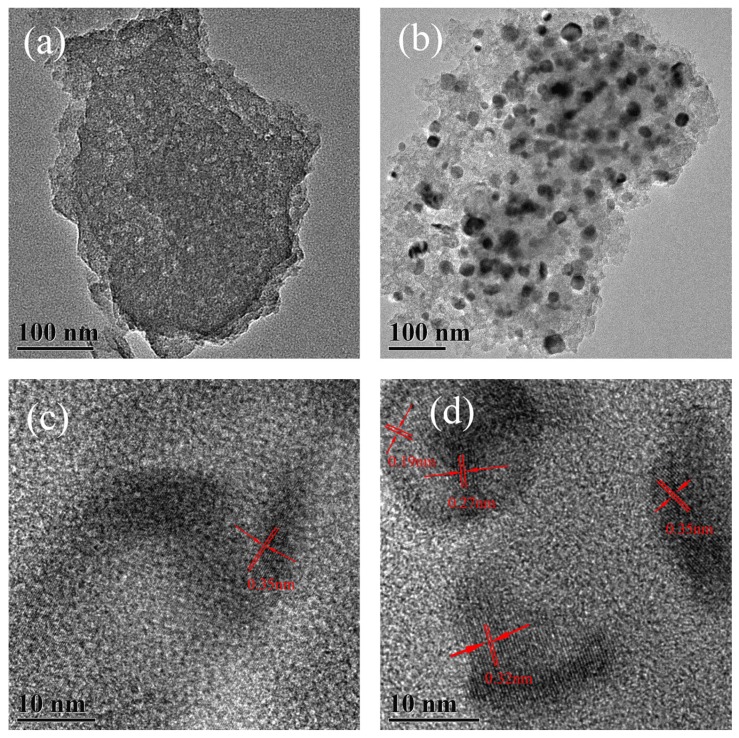
TEM images of MC (**a**) and MC–CeNPs (**b**), high resolution transmission electron microscopy (HRTEM) images of MC (**c**), and MC–CeNPs (**d**).

**Figure 3:**
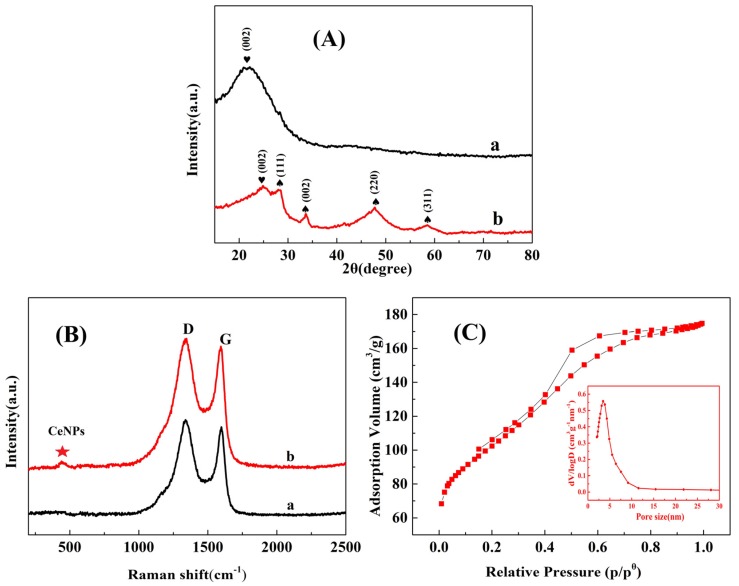
(**A**) XRD pattern of MC (**a**), MC–CeNPs (**b**); (**B**) Raman spectra of MC (**a**), MC–CeNPs (**b**); and, (**C**) N_2_ adsorption–desorption isotherm and pore size distribution (inset) of MC–CeNPs.

The dispersion state and microstructure were further investigated by TEM. Figure 2a shows MC with stacked thin sheets structure with lots of pores. CeNPs were dispersed uniformly on the MC sheets (Figure 2b), suggesting that MC could well act as a support for CeNPs, and an embedded structure was formed. An HRTEM image (Figure 2c) depicts the lattice fringes of MC exhibiting interplanar spacing of 0.35 nm [32], which was attributed to the graphite carbon lattice distance (002) [33]. Moreover, an HRTEM image of the MC–CeNPs (Figure 2d) exhibited the clear lattice fringes (111), (200), and (220) of cubic phase ceria with interplanar spacing of 0.33, 0.27, and 0.19 nm [34], respectively. The crystalline structures of the materials were checked using XRD measurement. In Figure 3A, curve (a) showed the diffraction peak of the graphite (002) at 2θ = 23.6° [35]. In addition, curve (b) exhibited the diffraction peaks at 2θ = 29.2°, 33.7°, 48.1°, and 56.9°, attributed to (111), (200), (220), and (311) planes of the cubic fluorite crystal structure of CeO_2_ (JCPDS 81-0792) [36], and no impurity was detected. The result corresponded with TEM, both proved that the MC–CeNPs were prepared successfully and they retained a well-defined crystal structure.

The Raman spectra of the MC (a) and MC–CeNPs (b) are shown in Figure 3B. The Raman spectra of MC exhibited two prominent peaks (D and G bonds) for graphite carbon (1338 and 1398 cm^−1^) [37]. In addition, a new peak in 457 cm^−1^ could be observed for MC–CeNPs, which was attributed to the F_2g_ vibration of the anchored CeNPs [38]. Nitrogen adsorption–desorption isotherms and the pore size distribution curve of MC–CeNPs are shown in Figure 3C. It could be found that the curve slowly increases at low relative pressure of 0.10–0.40, then a sharp increase occurs in the range of 0.40–0.80, which gives the type–IV shape [39]. Relevantly, the existence of mesopores is supported by the hysteresis of the desorption curve and the sharp increase in the adsorption capacity in the medium relative pressure region. Besides, Brunauer–Emmett–Teller (BET) measurements revealed that the specific surface area of MC–CeNPs was 362.4 m^2^·g^−1^ and the clear pore size distribution was concentrated on 3.5 nm.

**Figure 4:**
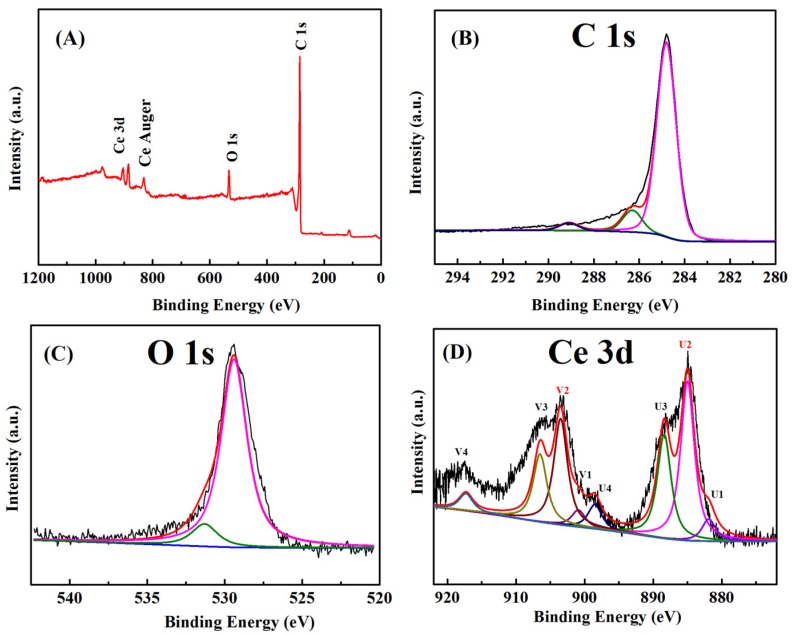
X-ray photoelectron spectroscopy (XPS) spectra of MC–CeNPs: (**A**) the survey spectrum; (**B**) C1s peak; (**C**) O1s peak; and, (**D**) Ce 3d peak.

XPS was employed to analyze the MC–CeNPs composition and the binding state of the elements. The XPS survey of the nanocomposite (Figure 4A) demonstrates three elements Ce, C, and O in the composites; the peak appearing at 831.7 ev belongs to the Auger peak of Ce. As shown in Figure 4B, there were three peaks centered at 284.8, 286.3, and 289.3 eV in the C 1s XPS spectrum, which assigned to C=C, C–O, and C=O [40]. The O1s spectrum (in Figure 4C) exhibited two peaks at 529.6 and 531.6 eV, which were respectively ascribed to the oxygen species of CeO_2_ and the adsorbed OH^-^ species on the surface [41]. The spectrum of Ce 3d was assigned to the 3d_5/2_ state (labelled U) and the 3d_3/2_ state (labelled V). The Ce 3d_3/2_ displayed four peaks at 882.3, 885.1, 888.4, and 898.6 eV, while 3d_5/2_ showed four other peaks at 901.1, 904.8, 907.4, and 916.8 eV (in Figure 4D) [42]. The peaks at 885.1 (U2) and 904.8 (V2) eV belonged to Ce^3+^, and the other peaks were ascribed to Ce^4+^, suggesting the existence of Ce^3+^ in MC–CeNPs with oxygen vacancies. This might be ascribed to CeNPs interacting with the MC sheets, and with a certain amount of Ce^4+^ turned into Ce^3+^ and some oxygen vacancies possibly produced. 

### 2.2. Electrochemical Behavior of the Modified Electrodes 

**Figure 5:**
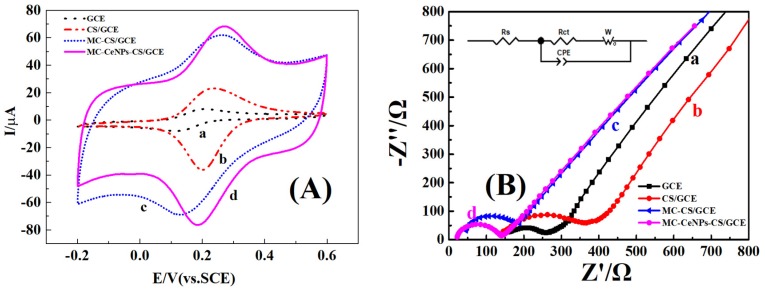
Cyclic voltammetry (CV) (**A**) and electron impedance spectroscopy (EIS) results (**B**) for glassy carbon electrode (GCE) (**a**), CS/GCE (**b**), MC–CS/GCE (**c**), MC–CeNPs–CS/GCE (**d**) in 1 mM K_3_Fe(CN)_6_/K_4_Fe(CN)_6_ containing 0.1 M KCl. Scan rate: 100 mV·s^−1^, Frequencies swept: 10^5^ to 0.01 Hz.

Cyclic voltammetry (CV) was applied to investigate the electrochemical responses of different modified electrodes in 1 mM K_3_Fe(CN)_6_/K_4_Fe(CN)_6_ containing 0.1 M KCl. As shown in Figure 5A, a higher pair of redox peaks was observed for MC–CeNPs–CS/GCE as compared with other electrodes, which indicated that MC–CeNPs–CS/GCE had the larger real area and more active sites [43]. Furthermore, the electron transfer capabilities of different modified electrodes were checked using electron impedance spectroscopy (EIS). Figure 5B shows the EIS curves of GCE (a), CS/GCE (b), MC–CS/GCE (c), and MC–CeNPs–CS/GCE (d), with a frequency range of 10^5^ to 0.01 Hz. The inset in Figure 5B exhibits the Randles equivalent circuit model [44]. In the equivalent circuit, R_ct_ is the electron–transfer resistance, R_s_ is the bulk electrolyte resistance, CPE is the interfacial capacitance, and W is the Warburg resistance, which is related to the diffusion of the electrolyte on the electrode surface. The semicircle in the higher-frequency regions reflects R_ct_ and the straight line in the lower-frequency region corresponds to the diffusion process [45]. The R_ct_ values of the different electrodes were in the order of MC–CeNPs–CS/GCE (119.7 Ω) < MC–CS/GCE (148.6 Ω) < GCE (182.4 Ω) < CS/GCE (301.4 Ω). The result indicated that the MC–CeNPs–CS/GCE had higher conductivity and a faster electron transfer process. 

### 2.3. Electrochemical Behaviors of HQ and CC

**Figure 6:**
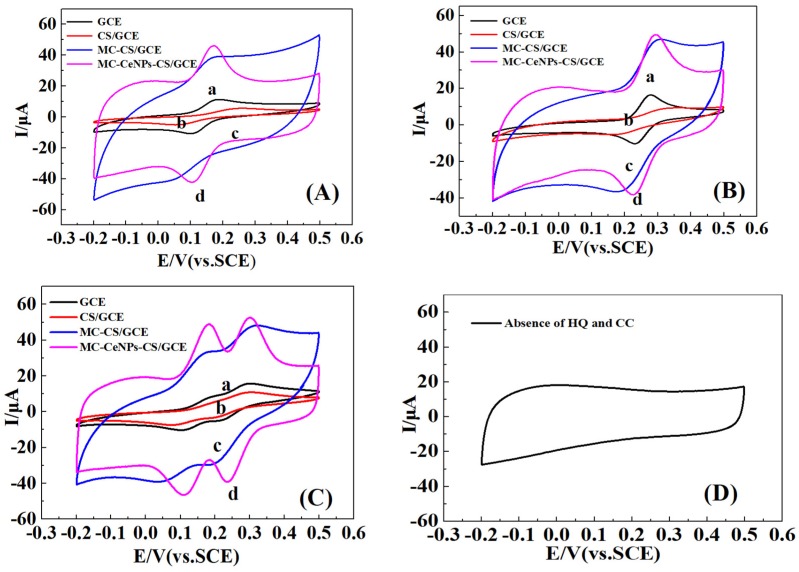
CV results of 0.2 mM HQ (**A**), 0.2 mM CC (**B**), and 0.2 mM HQ and CC (**C**) for the different modified electrodes in pH 5.0 CBS at a scan rate of 0.1 V s^−1^. (**D**) MC–CeNPs–CS/GCE in the absence of HQ and CC.

To investigate the potential applications of the different materials, the electrochemical behaviors of HQ and CC were also studied through the CV method. Figure 6A–C show the CV responses of GCE (a), CS/GCE (b), MC–CS/GCE (c), MC–CeNPs–CS/GCE (d) in 0.1 M CBS (pH 5, containing 0.2 mM of HQ, CC, and their mix solution) at 0.1 V s^−1^(E: −0.2 to 0.5 V (vs. SCE)), respectively. It can be observed that the CS/GCE exhibited the poorest redox peaks in all solutions. Furthermore, the current peaks of the independent presence of HQ and CC on GCE were relatively obvious, but they overlapped in the mix. However, the peak currents that were obtained from the MC–CS and MC–CeNPs–CS modified electrodes increased significantly, and the redox peaks were easy to distinguish. Especially, MC–CeNPs–CS/GCE exhibited well-defined redox currents and selectivity for the analytes, owing to the excellent conductivity, large specific surface area, and prominent catalytic ability of MC–CeNPs. In addition, the behavior of MC–CeNPs–CS/GCE in the absence of HQ and CC was investigated (Figure 6D) and no redox peak was observed, which demonstrated MC–CeNPs–CS did not interfere with the experiment and could be a suitable material for the determination of HQ and CC. 

### 2.4. Effect of pH

**Figure 7:**
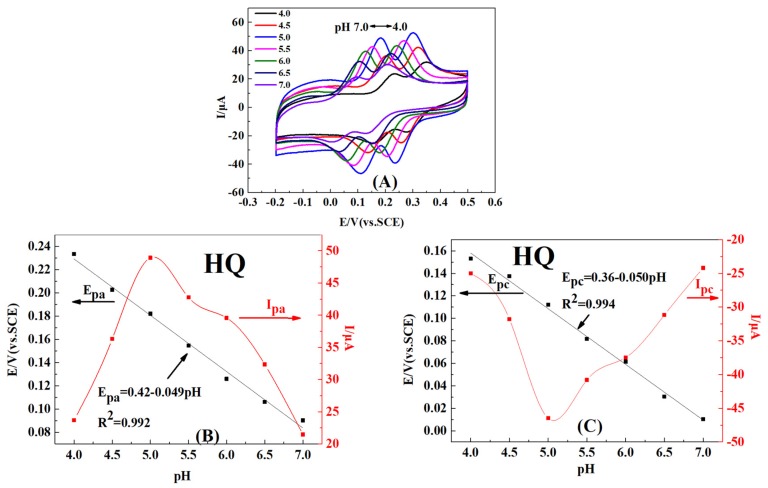

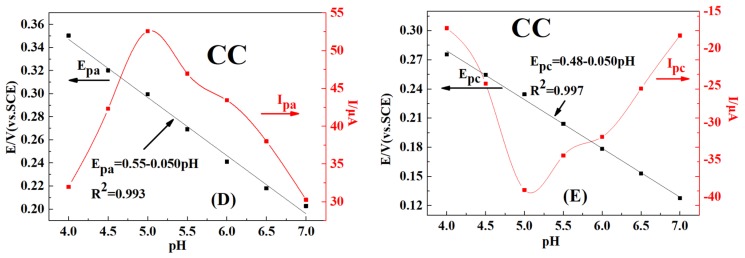
(**A**) CV results of MC–CeNPs–CS/GCE with 0.2 mM HQ and CC in CBS at different pH values (scan rate 0.1 V s^−1^). (**B**,**C**) The relationship of E_p_ (the black line region) and I_p_ (the red line region) with pH for HQ. (**D**,**E**) The relationship of E_p_ (the black line region) and I_p_ (the red line region) with pH for CC.

The effect of pH on the simultaneous determination of HQ and CC was studied via CV. As depicted in Figure 7A, the CV response of 0.2 mM HQ and CC was recorded; in 0.1 M CBS with different pH values (4.0 to 7.0) at scan rate of 0.1 V s^−1^; the cathode peak potential (E_pc_) and anode peak potential (E_pa_) shifted negatively, indicating that protons were involved in the electrochemical redox process [46]. Furthermore, the E_pa_ and E_pc_ exhibited a linear relationship with increasing pH, expressed as E_pa_ (V) = 0.42–0.049 pH (R^2^ = 0.992) and E_pc_ (V) = 0.36–0.050 pH (R^2^ = 0.994), E_pa_ (V) = 0.55–0.050 pH (R^2^ = 0.993), and E_pc_ (V) = 0.48–0.050 pH (R^2^ = 0.997) for HQ and CC, respectively. In addition, these slopes were close to the ideal Nernst theoretical value (0.059 V, 25 °C), implying that an equal amount of proton and electron transfer occurred in the electrochemical reaction [47].

In addition, it can be seen from Figure 7B–E that the current signal (the cathode peak current I_pc_ and oxide peak current I_pa_) first increased and reached its maximum value at pH 5 and then decreased with a further increase of the pH value. The pK_a_ values of HQ and CC are 9.85 and 9.4; at higher pH, these two molecules were deprotonated and converted to anions. Furthermore, the functional groups of CS (–NH_2_) also became deprotonated, which caused the modified electrodes to possess negative charges. Thus, the electrostatic repulsion between electrode and analytes would decrease the adsorption on the electrode. Consequently, the peak currents decreased with increasing pH value. The maximum current responses of HQ and CC appeared at pH 5, hence pH 5 was selected as an optimum condition for the following experiment.

### 2.5. Effect of Scan Rate 

**Figure 8:**
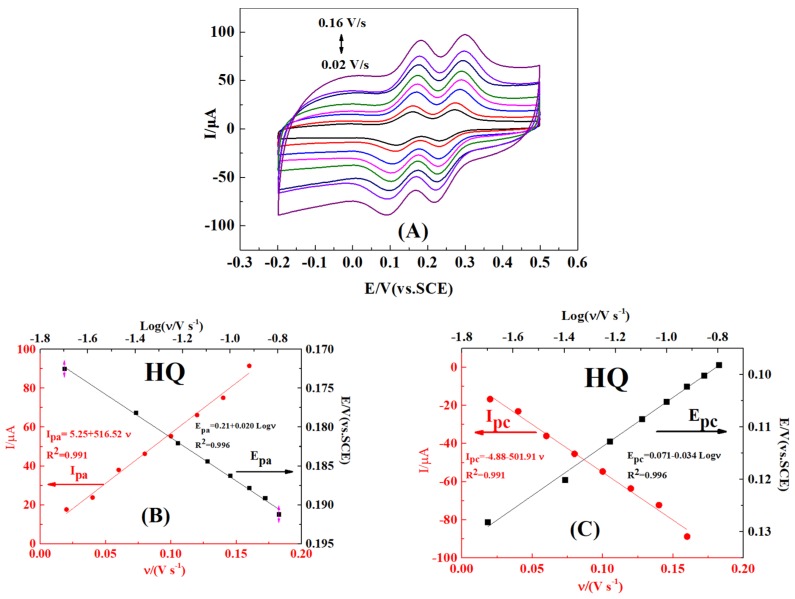

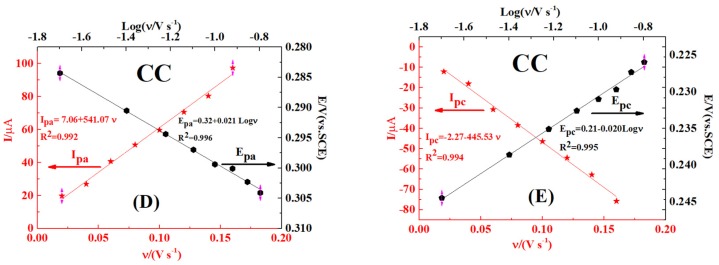
(**A**) CV results of 0.2 mM HQ and CC on MC–CeNPs–CS/GCE in 0.1 M CBS (pH 5.0) with different scan rates (0.02–0.16 V s^−1^). (**B**,**C**) Plots of I_p_ (the red line region) vs. ν and E_p_ (the black line region) vs. log ν for HQ. (**D**,**E**) Plots of I_p_ (the red line region) vs. ν and E_p_ (the black line region) vs. log ν for CC.

To investigate the reaction mechanism of HQ and CC during the electrochemical process, the effect of scan rate was further studied via CV with different scan rates (0.02, 0.04, 0.06, 0.08, 0.1, 0.12, 0.14, 0.16 V s^−1^). As displayed in Figure 8A, it can be observed that the peak current signals of HQ and CC were enhanced with increasing scan rates, and that E_pa_ shifted positively and E_pc_ moved negatively.

Furthermore, Figure 8B–E (the red line region) show the linear relationship between I_p_ and increasing scan rates (ν), which can be expressed as I_pa_ = 5.25 + 516.52 ν (R^2^ = 0.991) and I_pc_ = 4.88–501.91 ν (R^2^ = 0.991) for HQ and as I_pa_ = 7.06 + 541.07 ν (R^2^ = 0.992) and I_pc_ = 2.27–445.53 ν (R^2^ = 0.994) for CC. These relationships indicate that the electrochemical process of HQ and CC on MC–CeNPs–CS/GCE is typical adsorption–controlled [48]. Laviron theory [49] was used to study the reaction mechanism:

(1) Ip = nFQν/4RT

where n is the number of electrons, F is the Faraday constant (96485 C mol^−1^), Q is the electric quantity, R is the universal gas constant (8.314 J K^−1^ mol^−1^), and T is the temperature in kelvin. The n can be calculated, the values are 1.69 and 1.85 for HQ and CC, indicating that the reaction of HQ and CC in modified electrode is complex, which is not a simple two–electron process [46,47,48].

In addition, Figure 8B–E (the black line region) depict that the relationship between E_p_ and the corresponding log ν. The linear regression equations were E_pa_ = 0.21 + 0.020 log ν (R^2^ = 0.996) and E_pc_ = 0.071–0.034 log ν (R^2^ = 0.996), E_pa_ = 0.32 + 0.021 log ν (R^2^ = 0.996) for HQ, and E_pc_ = 0.21–0.020 log ν (R^2^ = 0.995) for CC. The electrochemical parameters, such as charge transfer coefficient (α) and electron transfer coefficient (K_s_), were calculated by following Laviron’s eq [31,49]

(2) $$E_{\mathrm{pa}}{\;=\;E}^{\theta }\;+\left(\frac{2.3\mathrm{RT}}{\left(1-\alpha \right)\mathrm{nF}}\right)\log\;\nu \;$$

(3) $${\;E}_{\mathrm{pc}}{\;=\;E}^{\theta }–\left(\frac{2.3\mathrm{RT}}{\alpha \mathrm{nF}}\right)\log\;\nu \;$$

(4) $${\mathrm{logk}}_{s}\;=\;\alpha \log\left(1-\alpha \right)+\left(1-\alpha \right)\log\alpha -\log\left(\frac{\mathrm{RT}}{\mathrm{nFv}}\right)-\left(1-\alpha \right)\frac{\alpha \mathrm{nF}\Delta E}{2.3\;\mathrm{RT}}$$

Here, ΔE represents the potential separation between E_pc_ and E_pa_; the other notation is as previously defined. Thus, the values of α and k_s_ were estimated to be 0.45, 0.69 s^−1^ for HQ, and 1.09, 1.05 s^−1^ for CC. These k_s_ values are better than those for sensors that are based on CS [50,51], indicating that HQ and CC have faster electron transfer kinetics on MC–CeNPs–CS/GCE. 

### 2.6. Simultaneous Determination of HQ and CC 

**Figure 9:**
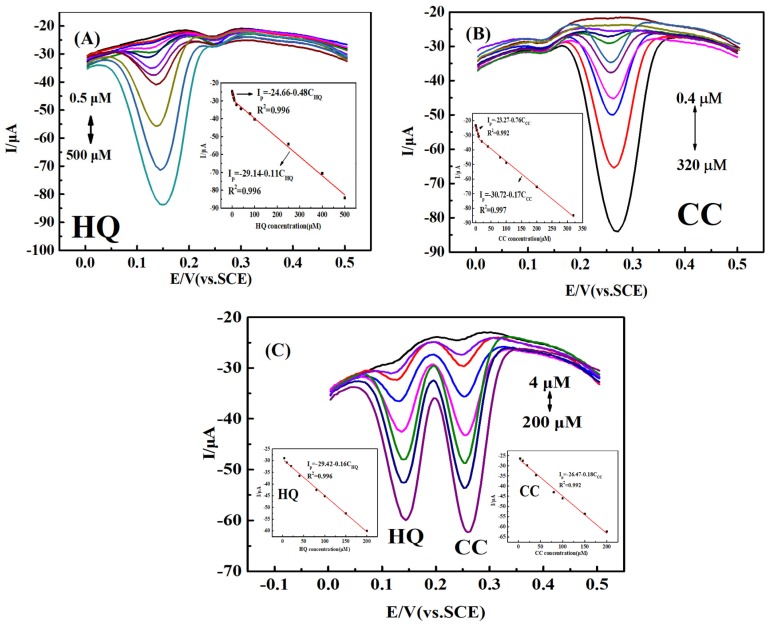
(**A**) Differential pulse voltammetry (DPV) results for different concentrations of HQ (0.5–500 µM) in the presence of 10 µM CC (**B**) DPV results for different concentrations of CC (0.4–320 µM) in the presence of 10 µM HQ. (**C**) DPV results for the simultaneous determination of CC and HQ of different concentrations (4–200 µM).

Differential pulse voltammetry (DPV) was carried out to record the peak currents of HQ and CC on MC–CeNPs–CS/GCE. The DPV responses for HQ and CC on MC–CeNPs–CS/GCE are displayed in Figure 9A,B, the independent determination of HQ and CC was obtained, while the concentration of one species was changed and the other was held constant. As shown in Figure 9A, the peak current increased with an increasing concentration of HQ in the presence of 10 μM CC. Furthermore, the current of CC remained almost constant, which indicated that the oxidation of HQ and CC on MC–CeNPs–CS/GCE took place individually. In addition, the peak current exhibited linear relationships with the concentration of HQ (0.5–10 μM and 10–500 μM): two linear calibration curves were obtained and expressed as I_p_ = −24.66–0.48 C_HQ_ (R^2^ = 0.996) and I_p_ = −29.14–0.11 C_HQ_ (R^2^ = 0.996). Similarly, Figure 9B depicts the DPV results of various concentrations of CC in the presence of 10 μM HQ, and the regression equations found were I_p_ = −23.27–0.76 C_CC_ (R^2^ = 0.992) and I_p_ = −30.72–0.17 C_CC_ (R^2^ = 0.997) for the C_CC_ ranges of 0.4–10 μM and 10–320 μM. The detection limits for HQ and CC were 0.24 µM and 0.13 µM (S/N = 3), respectively. A comparison between MC–CeNPs–CS/GCE and the other reported electrodes is shown in Table 1. Two linear ranges existed from low to high concentration for both HQ and CC. This could be attributed to MC–CeNPs–CS/GCE having limited active sites. During the electrochemical process, the analytes firstly reacted in highly active sites. When the highly active sites reached saturation, the analytes would be slowly enriched onto the other sites with low activity. Thus, the slope of low–concentration range was bigger than that of the high concentration range [52]. Moreover, the active sites were saturated gradually with increasing concentration, and there was no linear relationship with higher concentration. 

To further investigate the performance of the MC–CeNPs–CS/GCE, the DPV responses of HQ and CC were also studied, when the concentration of these two species increased simultaneously. As shown in Figure 9C, the current increased with the concentration from 4 to 200 µM. The regression equations were I_p_ = −29.42–0.16 C_HQ_ (R^2^ = 0.996) and I_p_ = −26.47–0.18 C_CC_ (R^2^ = 0.992). All of these results indicate that the sensor can be successfully used for the simultaneous determination of coexisting CC and HQ, without the species interfering with each other.

### 2.7. Reproducibility, Stability, and Selectively 

**Figure 10:**
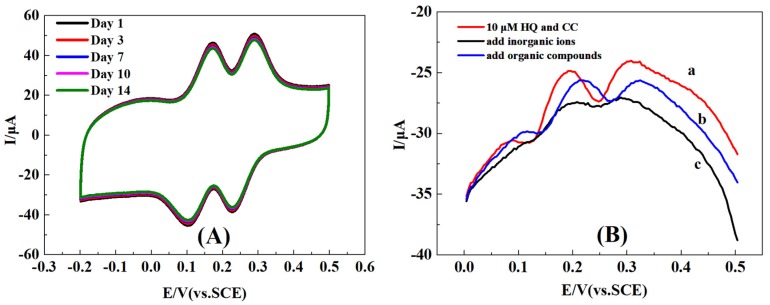
(**A**) CV results of 0.1 M CBS containing 200 µM HQ and CC recorded for 14 days. (**B**) DPV results of 0.1 M citrate buffer solution (CBS) containing 10 µM HQ and CC, (**a**) without any interfere, (**b**) adding inorganic ions, and (**c**) adding organic compounds.

The repeatability of the MC–CeNPs–CS/GCE was examined by monitoring the DPV response of 10 µM HQ and CC for five individual measurements. The relative standard deviations (RSDs) were 2.5% and 3.7% for HQ and CC, respectively. Moreover, the stability of MC–CeNPs–CS/GCE was also investigated by recording the change in CV plots over 14 days, in 0.1 M CBS containing 200 µM HQ and CC. As shown in Figure 10A, after 14 days, the current response remained about 96.4% of the original value. In addition, a selectivity study of the MC–CeNPs–CS/GCE was carried out by DPV in a mixed solution (10 µM HQ and CC) containing some inorganic ions and organic compounds. The result in Figure 10B shows that 100-fold Na^+^, K^+^, NH_4_^+^, Ca^2+^, Mg^2+^, Cu^2+^, Pb^2+^, Al^3+^, Fe^3+^, Cl^–^, NO_3_^–^,SO_4_^2−^, Br^-^, and I^-^, and 50–fold resorcinol, nitrophenol, bisphenol A, and phenol did not interfere with the determination (signal change below 5%). In summary, all of these results indicate that the sensor has acceptable reproducibility, long-term stability, and satisfactory anti–interference ability for the determination of HQ and CC. 

### 2.8. Real Sample Analysis

To explore the potential for practical application, tap water and lake water were selected as samples for quantitative analysis and tested by the standard addition method. Each sample was tested five times by five individually electrodes. The obtained average values and the RSDs are shown in Table 2. The recoveries were in ranges of 98.5–103.2% and 98–103.4% for HQ and CC, respectively, suggesting that this sensor has feasibility, reliability, and potential for the determination of HQ and CC in real samples. 

## 3. Experimental

### 3.1. Materials and Reagents

Pluronic F_127_ (EO_106_PO_70_EO_106_, Mw = 13,000) purchased from Shanghai Macklin Biochemical Co., Ltd., Shanghai, China. HQ, CC, chitosan (CS), tetraethyl orthosilicate (TEOS), Phenol, formalin (37 wt %), cerium nitrate hexahydrate (Ce(NO_3_)_3_·6H_2_O), citric acid (C_6_H_8_O_7_·H_2_O), and trisodium citrate dehydrate (Na_3_C_6_H_5_O_7_·2H_2_O) were provided by Sinopharm Chemical Reagent Co., Ltd. (shanghai, China). All of the chemical reagents were used without further purification. All of the solutions were prepared with ultrapure water (18.2 MΩ).

### 3.2. Synthesis of the MC–CeNPs Nanocomposite

The carbon source (resol phenolic resin) was prepared according to the method by Fan et al. [64]. The MC–CeNPs nanocomposite was synthesized via the EISA method. Briefly, 1.6 g F127 was added into the mix solution (8 g C_2_H_5_OH, 1 g 0.2 M HCl) at 40 °C and stirred for 0.5 h to obtain a clear solution. A quantity of 0.2 g Ce(NO_3_)_3_·6H_2_O was dissolved in 5 g resol phenolic resin to form a brown solution. The two solutions were mixed together, and 2.03 g TEOS was added with stirring for 2 h; this mix solution was poured into petri dishes to evaporate ethanol at room temperature, followed thermopolymerization at 100 °C. The products, brown or transparent membranes, were scraped from the dishes, then calcined at 350 °C for 3 h and 900 °C for 2 h in N_2_. The carbon–silica–ceria composite was immersed in HF (10 wt %) in air for 24 h to remove the silica. Finally, the MC–CeNPs nanocomposite was obtained after washing and drying. The MC was synthesized the same way but without Ce(NO_3_)_3_·6H_2_O. 

### 3.3. Preparation of Modified Electrode 

The glassy carbon electrode (GCE, d = 3 mm) was polished using 0.1 μM, 0.05 μM, and 0.05 μM Al_2_O_3_ to obtain a mirror-like surface, and then sonicated with acetone and ultrapure water for 5 min. To prepare the electrocatalytic suspension, 3 mg MC–CeNPs was dispersed in 1 mL CS solution (dissolved in 1 wt % acetic acid, 1 mg mL^−1^), and the solution was sonicated for 30 min, obtaining a black and homogeneous suspension. Subsequently, 10 µL of MC–CeNPs–CS solution was dropped onto pretreated GCE and dried using an infrared lamp. Thus, the MC–CeNPs modified electrode was prepared, denoted as MC–CeNPs–CS/GCE. The electrodes of MC–CS/GCE and CS/GCE were obtained through a similar method with the same dosage. 

### 3.4. Characterization 

Scanning electron microscope (SEM) images and energy dispersive X-ray detector (EDX) spectra were recorded on a field emission scanning electron microscope (Nova NanoSEM450, FEI Co., Hillsboro, OR, USA). Transmission electron microscopy (TEM) images and high resolution transmission electron microscopy (HRTEM) images were obtained using a JEOL 2100 transmission electron microscope (Tokyo, Japan). X-ray diffraction (XRD) patterns were recorded on a Bruker D4 X-ray diffractometer (Karlsruhe, Germany) with Ni–filtered Cu Kα radiation (40 kV, 40 mA). The Brunauer–Emmett–Teller (BET) surface area was calculated from 77 K N_2_ adsorption–desorption isotherms by NovaWin 1000e (Quantachrome, Boynton Beach, FL, USA) and the pore size distributions were derived using the Barrett–Joyner–Halenda (BJH) model. Raman spectra were recorded using a Raman spectrometer (Dong Woo 500i, Seoul, Korea). The X-ray photoelectron spectroscopy (XPS) measurements were performed on a Thermo ESCALAB 250 with Al Kα excitation (New York, NY, USA). 

### 3.5. Electrochemical Measurements

Electrochemical experiments were performed using a CS350H electrochemical workstation (Wuhan Corrtest Instrument Corp., Ltd., Wuhan, China) and a conventional three-electrode cell was used. The GCE or modified GCE was employed as the working electrode, a saturated calomel electrode (SCE), and a platinum wire electrode served as the reference electrode and counter electrode, respectively. The differential pulse voltammetry (DPV) parameters were as follows: potential range, 0–0.5 V; potential increment, 0.004 V; amplitude, 0.025 s, frequency, 5 Hz. The cyclic voltammetry (CV) measurements were carried out for a fixed potential range and scan rate. 

The electrolytes for the electrochemical characterization were 0.1 M KCl and 1 mM K_3_Fe(CN)_6_/K_4_Fe(CN)_6_ solutions. As for the determination of HQ and CC, the supporting electrolyte (citrate buffer solution (CBS)) was prepared using 0.1 M C_6_H_8_O_7_·H_2_O and Na_3_C_6_H_5_O_7_·2H_2_O; the pH was adjusted by their proportions.

All of the tests were carried out in the presence of oxygen.

## 4. Conclusions 

In this study, a simple and sensitive electrochemical method was established for the simultaneous detection of HQ and CC. The prepared nanostructure composite possessed a large specific surface area, clear pore size distribution, excellent stability, high conductivity, and superior electrocatalytic ability, which improved the adsorption of analytes on the surface of electrode and provided faster electron transfer. Additionally, the MC–CeNPs–CS/GCE exhibited excellent performance in the simultaneous detection of HQ and CC with a low detection limit, wide linear range, and high selectivity and long-term stability. The sensing platform was applied to detection in real water sample with satisfactory results.

## Figures and Tables

**Table 1:** Comparison of the simultaneous determination of HQ and CC by different electrodes.

Electrode	Linear Range (µM)		Detection Limit (µM)		Ref.
	HQ	CC	HQ	CC	
c–MWCNTs *^a^*/CTS *^b^*/Au/GCE	0.5–1500	5–900	0.17	0.89	[53]
Cu–MOF199 *^c^*/SWCNTs *^d^*/GCE	0.1–1453	0.1–1150	0.08	0.1	[54]
Ni/N–MWCNT *^e^*	0.1–301	0.1–301	0.01	0.01	[55]
CS/MWCNTs/PDA *^f^*/AuNPs *^g^*/GCE	0.1–10	0.1–10	0.035	0.047	[56]
Cu_3_(btc)_2_ *^h^*/CS–ERGO *^i^*	5.0–400	2.0–200	0.017	0.069	[57]
CS/f–MWCNT *^j^*	0.09–171.4	0.09–155.4	0.027	0.029	[58]
NiO/CNT/GCE	10–500	10–400	2.5	2.5	[59]
NCNF *^k^*–GCE	1–400	1–400	0.3	0.4	[60]
CNCs *^l^*–RGO/GCE	1–400	1–300	0.87	0.40	[61]
BG *^m^*	5–100	1–75	0.3	0.2	[62]
P–rGO *^n^*	5–90	5–120	0.08	0.18	[63]
MC–CeNPs–CS/GCE	0.5–10,	0.4–10,	0.24	0.13	This work
	10–500	10–320			

Abbreviations: *^a^* carboxy–functionalized multi–walled carbon nanotubes; *^b^* chitosan; *^c^* metal–organic frameworks; *^d^* single–walled carbon nanotubes; *^e^* nitrogen–doped multiwalled carbon nanotubes; *^f^* polydopamine; *^g^* glod nanoparticles; *^h^* benzene–1,3,5–tricarboxylic acid; *^i^* electrochemically reduced graphene oxide; *^j^* (CS)–stabilized multi–walled carbon nanotube; *^k^* nitrogen–doped carbon nanofiber; *^l^* carbon nanocages; *^m^* Boron–doped grapheme; *^n^* porous reduced graphene oxide.

**Table 2:** Determination of CC and HQ in tap water and lake water samples using MC–CeNPs–CS/GCE by DPV.

Sample	Added( μM)		Found( μM)		Recovery (%)		RSD (%)	
	HQ	CC	HQ	CC	HQ	CC	HQ	CC
Tap water	0	0	0	0	–	–	–	–
	10	10	9.9	9.5	99.0	98.6	2.3	1.2
	50	50	51.6	51.7	103.2	103.4	3.6	2.7
	100	100	102.7	100.7	102.7	100.7	2.8	3.4
	0	0	0	0	–	–	–	–
Lake water	10	10	10.1	9.8	101.0	98.0	1.4	3.6
	50	50	49.8	50.6	99.6	101.2	3.1	2.9
	100	100	98.5	99.2	98.5	99.2	1.9	2.6
